# Haplotype Inference Using Long-Read Nanopore Sequencing: Application to *GSTA1* Promoter

**DOI:** 10.1007/s12033-024-01213-7

**Published:** 2024-06-17

**Authors:** Vid Mlakar, Isabelle Dupanloup, Yvonne Gloor, Marc Ansari

**Affiliations:** 1 CANSEARCH Research Laboratory, Geneva University Medical School, Rue Michel Servet 1, 1211 Geneva, Switzerland; 2 Swiss Institute of Bioinformatics, Lausanne, Switzerland; 3 Onco-Hematology Unit, Pediatric Department, Geneva University Hospital, Rue Willy-Donzé 6, 1205 Geneva, Switzerland

**Keywords:** GSTA1, Nanopore, Sequencing, Haplotypes, Phasing

## Abstract

Recovering true haplotypes can have important clinical consequences. The laboratory process is difficult and is, therefore, most often done through inference. In this paper, we show that when using the Oxford nanopore sequencing technology, we could recover the true haplotypes of the *GSTA1* promoter region. Eight LCL cell lines with potentially ambiguous haplotypes were used to characterize the efficacy of Oxford nanopore sequencing to phase the correct GSTA1 promoter haplotypes. The results were compared to Sanger sequencing and inferred haplotypes in the 1000 genomes project. The average read length was 813 bp out of a total PCR length of 1336 bp. The best coverage of sequencing was in the middle of the PCR product and decreased to 50% at the PCR ends. SNPs separated by less than 200 bp showed > 90% of correct haplotypes, while at the distance of 1089 bp, this proportion still exceeded 58%. The number of cycles influences the generation of hybrid haplotypes but not extension or annealing time. The results demonstrate that this long sequencing reads methodology, can accurately determine the haplotypes without the need for inference. The technology proved to be robust but the success of phasing nonetheless depends on the distances and frequencies of SNPs.

## Introduction

One single nucleotide polymorphism (SNPs) can have a considerable influence on gene expression and/or protein activity. However, several polymorphisms can affect the same target and their combined influence may determine the abundance and functionality of the resulting gene product. Such SNP combinations define haplotypes that tend to be inherited together. Pharmaco-genes encode enzymes which are important for the metabolism of exogenous compounds, including many drugs on the market. Several clinically important haplotypes affecting drug metabolic activity have already been identified and the establishment of corresponding drug dose adjustment guidelines has been proven beneficial to the patients [1–3].

Glutathione *S*-transferases are enzymes involved in the clearing of carcinogens, therapeutic drugs, environmental toxins, and toxic endogenous products by conjugation with glutathione [4, 5]. Although there are many classes of GSTs, the most abundant enzyme of this family found in the liver is GSTA1 [6, 7]. GSTA1 is involved in the clearance of important chemotherapeutic drugs including thiotepa [8, 9], doxorubicin [10], cyclophosphamide [11], and busulfan [12]. We previously demonstrated that six polymorphisms in the promoter region of *GSTA1* are associated with busulfan clearance [13, 14]. These polymorphisms are found in different haplotype and diplotype combinations which modulate the expression of *GSTA1* and influence the metabolic capacity of the cells [15]. Individuals can be stratified into three different metabolizer groups, slow, normal, and rapid metabolizers, based on their genotypes, to help personalize busulfan dose administration [15].

Up to now, haplotypes in the *GSTA1* promoter region are mostly determined from non-allele discriminating double-stranded DNA sequences by inference based on linkage disequilibrium between different polymorphisms reported in knowledge databases such as the 1000 genomes project [15]. However, this approach can lead to ambiguous results if two possible haplotype combinations result in the same diplotype or even erroneous conclusions in the case of previously non-recognized combinations [15–17]. Therefore, more accurate methods for haplotype phasing and allele discrimination have been developed to improve clinical diagnostics. However, the techniques available so far involve either time-consuming molecular cloning or the sequencing of several close relatives, if available [18]. These inherent difficulties considerably increase the workload and time-lapse to obtain the results which makes true phasing often impractical in routine medical practice. With the advent of next-generation technologies for DNA analysis, sequencing of single DNA molecules and phasing has become more accessible. However, standard NGS sequencing platforms, such as Illumina, produce short reads that still hamper haplotype phasing. Recently, Oxford Nanopore introduced a new platform supporting long reads single DNA molecule sequencing able to address this problem. The Nanopore technology, reviewed by Midha et al*.* [19], has already been successfully applied to the phasing of HLA and CYP2D6 alleles [20, 21]. Other long-read sequencing technologies, PacBio’s SMRT sequencing [22] and single-tube Long Fragment Read (stLFR) using DNA co-barcoding [23] could also be used for phasing because of the ability to read long reads on a level of a single DNA molecule. Despite the promising outlook of SMRT sequencing and stLFR, the Oxford Nanopore presents currently the best alternative considering the sequencing fidelity, cost, and labor to produce phase SNPs.

In this paper, we apply the Oxford nanopore sequencing technology to recover the true haplotypes of the *GSTA1* promoter region using a straightforward methodology. We propose a strategy accessible to most laboratories that would be applicable to other genomic regions where haplotype phasing is needed.

## Methods

### Samples and DNA Extraction

We selected cell lines from Coriell Institute (USA) for their high heterozygosity at our SNPs of interest according to the 1000 Genomes phase 3 data [24] (Table 1). DNA was extracted from the LCL cell lines using a DNeasy Blood & Tissue Kit (QIAgen, Germany) according to the manufacturer’s recommendations. LCLs were cultured in Roswell Park Memorial Institute Medium (RPMI) 1640 medium (Gibco, Carlsbad, CA) supplemented with 10% fetal bovine serum (HyClone, South Logan, UT) and 1% penicillin–streptomycin (Gibco) and incubated at 37 °C, 5% CO_2_-humidified atmosphere according to the manufacturer’s recommendations. The cell pellets for DNA extraction were harvested from cells passaged less than ten times [25].

**Table 1:** LCL cell line genotypes with inferred haplotypes from the 1000G data, genotype and diplotype phasing from nanopore sequencing data

Cell line	Inferred haplotype from the 1000G data	rs3957356 -52	rs3957357 -69	rs11964968 -513	rs4715332 -567	rs4715333 -631	rs58912740 -1142	Haplotype determined from Nanopore
NA06985	*A1*B1a	G|A	C|T	A|A	T|G	T|G	C|G	*A1*B1a
NA07056	*A1*B1a	G|A	C|T	A|A	T|G	T|G	C|G	*A1*B1a
NA07357	*A1*B1b	G|A	C|T	**A|G**	T|G	T|G	C|G	*A1*B1b
NA11840	*A1*B1a	G|A	C|T	A|A	T|G	T|G	C|G	*A1*B1a
NA12043	*A2*B1a	G|A	C|T	A|A	T|G	**G|G**	C|G	*A2*B1a
NA12762	*A1*B1a	G|A	C|T	A|A	T|G	T|G	C|G	*A1*B1a
NA12872	*A1*B1a	G|A	C|T	A|A	T|G	T|G	C|G	*A1*B1a
NA12874	*A1*B1a	G|A	C|T	A|A	T|G	T|G	C|G	*A1*B1a

### PCR for Amplification of GSTA1 Promoter Region, Sanger Sequencing, and Diplotype Determination

The *GSTA1* promoter region encompassing the six SNPs defining metabolizer classification was amplified using Platinum SuperFi II PCR master mix (Thermo Scientific, USA) with GSTA1-1336-F 5′-TGGATCCCTCAGTTTTGTAAGG-3′ forward and GSTA1-1336-R 5′-TAAACGCTGTCACCGTCC-3′ reverse primers (Microsynth, Switzerland) at a final concentration of 0.8 μM and 20 ng of DNA in final reaction volume of 20 μL. Standard *GSTA1* promoter PCRs were performed under the following cycling conditions: Initial denaturation at 95 °C for 3 min, 40 cycles of denaturation at 95 °C for 30 s, annealing at 64 °C for 30 s, extension at 72 °C for 45 s, and final extension at 72°C for 1 min.

For the testing of different PCR conditions on phasing, the duration of extension at 72 °C was decreased to 35 s, 25 s, or 15 s while maintaining the annealing at 30s. Next, we also decreased annealing time to 20 s and 15 s at 15 s extension time. Finally, we changed PCR cycles from 40 to 35, 30, 25, 20, and 15 at 20 and 120 ng of genomic DNA at each condition and at 30 s annealing and 45 s extension time. The PCR reactions were analyzed using Tapestation (Agilent, USA) with D5000 screen tape (Agilent, USA).

The genotype of all cell lines was confirmed with standard Sanger sequencing (Fasteris, Switzerland) using the PCR products produced with 20 ng of DNA and annealing and extension for 30 and 45 s, respectively, and the following primers GSTA1-1336-F, GSTA1-1336-R, and GSTA1_seq-52_F (5′-TGACGCAAAGAGGATAGCAT-3′).

The definition of the *GSTA1* promoter haplotypes and their corresponding star allele nomenclature is described in a previous work [15]. Reference *A1/*A1 is assigned by default to sequences without mutations at any of the 6 SNPs tested.

### Oxford Nanopore Sequencing and Analysis

Sequencing was performed using Nanopore sequencing (Microsynth, Switzerland). 25 ng of PCR products was used for each sequencing reaction.

Guppy version 6.4.6. was used for base calling, demultiplexing, and trimming of the barcodes (Oxford Nanopore Technologies Ltd., 2000). Quality control and summary reports for nanopore reads were generated using nanoq version 0.10.0 [26]. The quality of sequencing reads was evaluated using FastQC version 0.12.1 [27]. The reads were aligned to the reference sequence of chromosome 6 in the human genome (GRCh38 assembly) using MiniMap2 [28], with our *GSTA1* promoter region PCR extending from genomic coordinate 52803791 to 52805125 (GRCh38.p14, Chr:6, *GSTA1*). Variant calling was performed with clair3 [29] and haplotype phasing with WhatsHap [30].

### Estimating the Proportion of Reads Carrying True Haplotypes

We quantified the proportion of reads carrying the correct haplotypes for all pairs of heterozygous SNPs in the *GSTA1* promoter region. Briefly, we first extracted the corresponding nucleotides from the multiple alignments generated with minimap2 and sorted them with samtools version 1.17, using pysam [31]. We then estimated the proportion of reads that carried the correct haplotypes for pairs of variants using a series of steps in R version 4.3.2 [32]. Briefly, we loaded into R the reads ids with the carried nucleotide at each SNP location, merge all possible combinations and estimated the proportion of correct haplotypes out of all observed haplotypes. Before estimating this proportion, we discarded the reads carrying a nucleotide whose frequency was below 1/3 of the total number of reads covering that position.

## Results

### Nanopore Sequencing and Haplotype Phasing

**Fig. 1:**
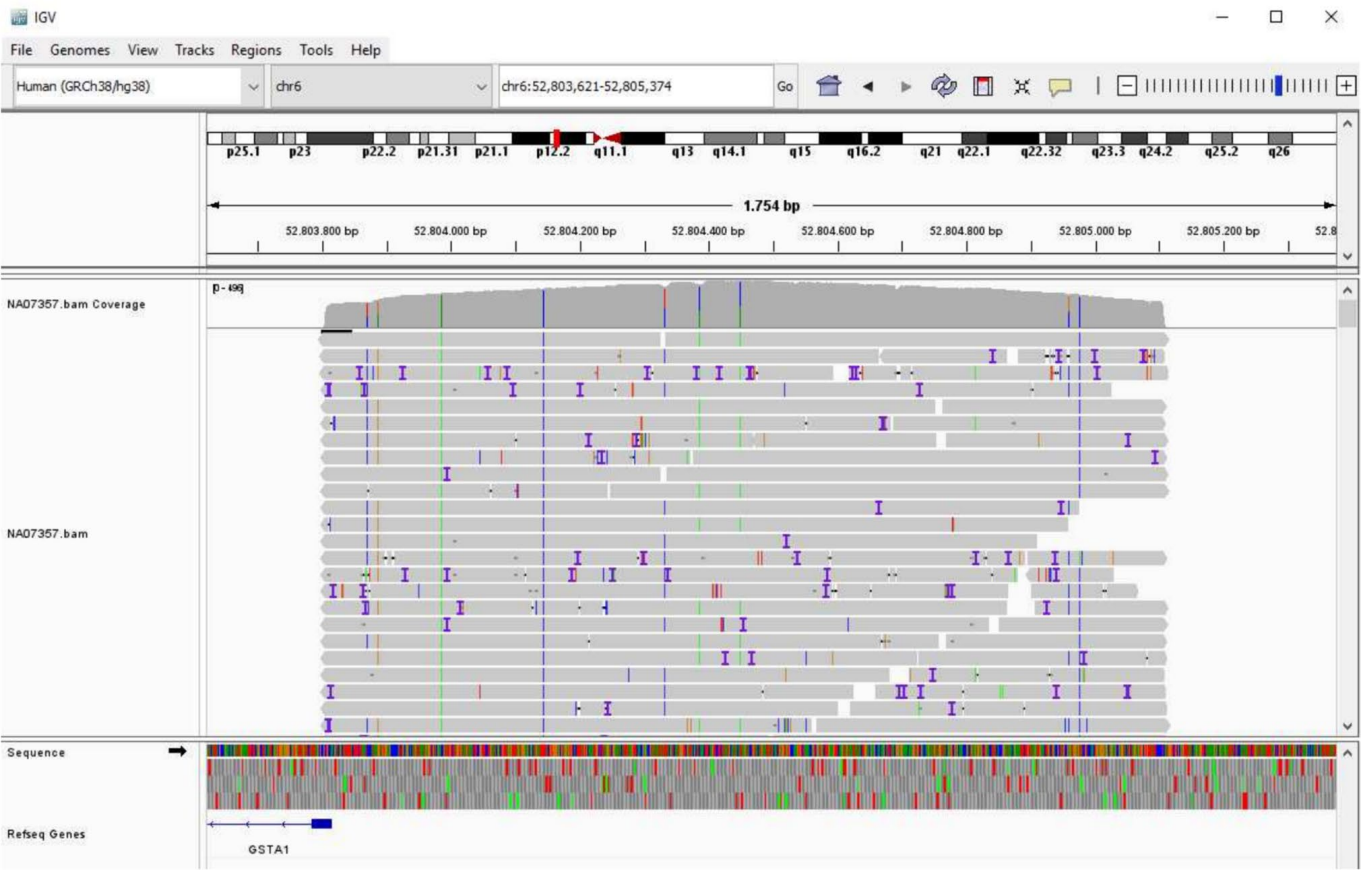
Example of Nanopore sequencing result alignment. The figure shows the alignment to the GSTA1 promoter region in the GRCh38 reference genome of the reads obtained from Nanopore sequencing for one LCL cell line (NA07357). The view was obtained with the Integrative Genomics Viewer (IGV). The horizontal gray bars correspond to sequencing reads. The colored bars show differences between the nucleotide carried by reads and the corresponding base in the reference genome

**Fig. 2:**
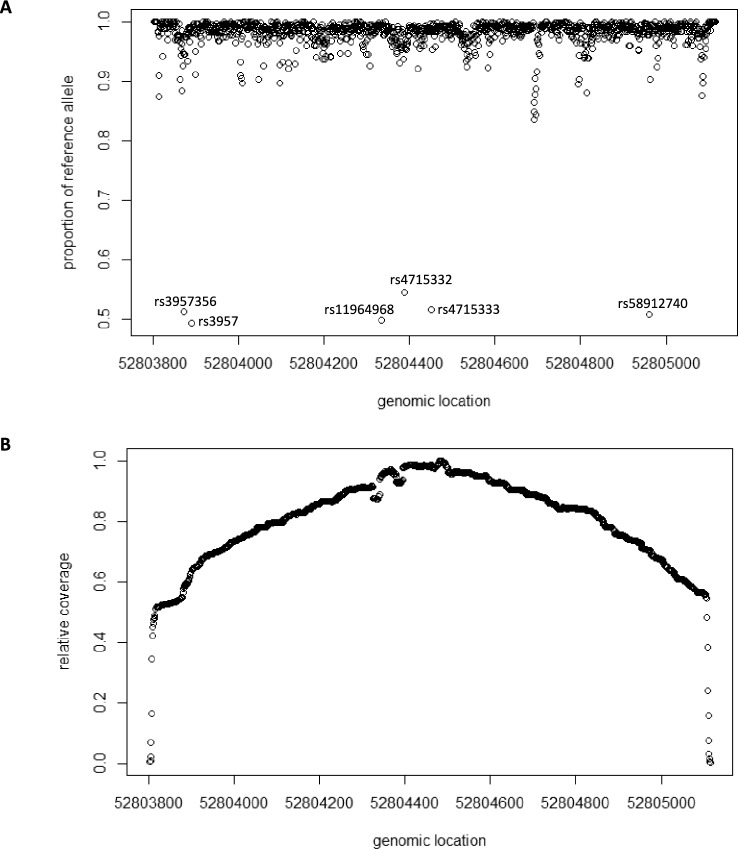
Control quality for Nanopore sequencing data. **A** Proportion of reads carrying the reference allele (NA07357). **B** Relative coverage over the genomic region (GRCh38.p14, Chr:6, 52803791 to 52805125) under investigation (NA07357)

On average, we sequenced 820 reads per sample, with an average length of 813 bp, which correspond to 60.9% of the length of the PCR product (i.e., 1336 bp) (Table 2). A large proportion of the reads (> 98%) were mapped to the genomic reference sequence of the *GSTA1* promoter region (Table 2). We show in Fig. 1 an example of mapping for the sequencing reads corresponding to one of the samples. We see a random distribution of the sequencing errors (Figs. 1, 2A) in the genomic region of interest. Also, we see 100% sequence coverage in the middle of the PCR product with the coverage decreasing to roughly 50% at the 5′ and 3′ of the PCR product (Fig. 2B).

**Table 2:** Oxford nanopore sequencing read mapping statistics

Cell line	Number of reads	Read length: mean and range in bp	% from total length	Number of reads mapped	% of reads mapped
NA06985	1039	822.6 [37–9184]	61.5	1031	99.23
NA07056	505	789.4 [136–9235]	59.1	502	99.41
NA07357	664	813.0 [34–5462]	60.9	656	98.80
NA11840	693	821.4 [43–6625]	61.5	648	93.51
NA12043	881	816.9 [127–5266]	61.1	862	97.84
NA12762	1108	812.3 [40–9171]	60.9	1090	98.38
NA12872	776	799.2 [83–4626]	59.8	766	98.71
NA12874	892	835.5 [43–9417]	62.5	880	98.65
Mean	820	813.8	60.9	804	98.05

The results obtained from the Nanopore sequencing were in complete agreement with the ones from the Sanger sequencing and 1000 genomes data (Table 1).

### Estimating the Proportion of Reads Carrying True Haplotypes

**Fig. 3:**
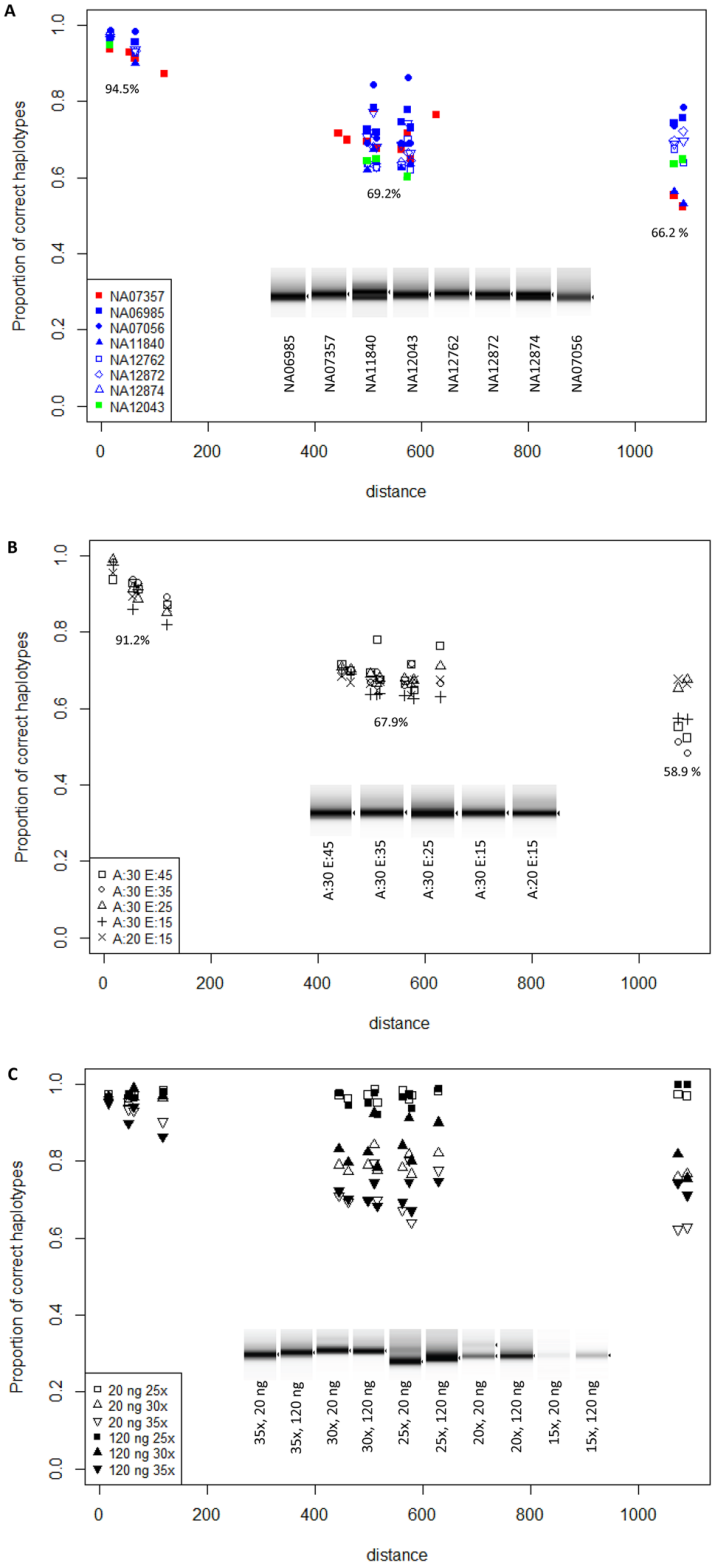
Phasing quality control. Proportions of reads with correct pairwise phasing for heterozygous SNPs in the *GSTA1* promoter region. In **A**, we show how this proportion varies according to the distance between SNPs and genotypes: *A1*B1b are shown in red, *A1*B1a in blue, and *A2*B1b in green. In **B**, we show how this proportion varies according to the distance between SNPs and PCR conditions for the NA07357 cell line. We prepared a range of PCR products by gradually decreasing the annealing time (A) from 45 to 35 s, 25 s, or 15 s at a fixed extension (E) time of 30 s. We also decreased the extension time (E) from 30 to 20 s at the 15 s annealing time. In **C**, we show how DNA content and number of cycles influence the presence of hybrid haplotypes. We analyzed PCR products of 25, 30, and 35 cycles because they produced enough DNA for analysis. PCRs generated with 25 cycles produced fewer hybrid haplotypes than those with 30 or 35 cycles. Initial DNA content has little influence on the amount of hybrid haplotypes

We estimated the proportion of sequencing reads carrying the true combination of variants. We show that this proportion decreases as a function of the distance between two loci (Fig. 3A). For a distance below 200 bp, > 91% of the reads carry the correct SNPs combination. This proportion still exceeds 58% at a distance of 1′089 bp.

### Influence of PCR Quality on Phasing

We next sought to assess the impact of PCR reaction conditions on phasing using Oxford nanopore sequencing. Indeed, non-fully extended PCR products might act as pseudo primers and anneal to either allele in the next cycle, resulting in hybrids producing sequences containing artificial haplotypes. To evaluate the risk of error resulting from the generation of hybrids, we prepared a range of PCR products by altering the extension (E) and the annealing (A) times (Fig. 3B) and the altering number of cycles and content of DNA (Fig. 3C). The analysis by TapeStation showed no effect on the overall quality of the PCR products when changing reaction conditions. We observed the influence of the number of cycles and the initial quantity of DNA on the final PCR quantity. Fifteen and twenty cycles did not produce enough DNA for Nanopore sequencing. The analysis of Nanopore sequencing showed no effect of extension and annealing time on the production of hybrid haplotypes. On the other hand, the number of cycles had a significant impact on the presence of hybrid haplotypes. Taken together, for a robust PCR reaction, the duration of annealing and extension time did not affect the frequency of crossovers found in the PCR products (Fig. 3B); however, the number of cycles did (Fig. 3C).

## Discussion

Here, we present a new strategy for phasing the *GSTA1* promoter haplotypes. We show that when using the Nanopore long-range sequencing technology, we could accurately determine the haplotypes of all tested samples and demonstrate the robustness of the technique for this specific application. In the case of *GSTA1* promoter genotyping, proper allelic discrimination is particularly important for the *A1*B1a/*A3*B2 haplotype pair that cannot be distinguished by standard Sanger sequencing. The results of Nanopore phasing confirmed that all six ambiguous samples carried the *A1*B1a genotype in agreement with the rare occurrence of the *A3 haplotype in human populations. GSTA1*A3 has been observed only in the Indian population of Gujarati at the frequency of 0.005. The haplotypes *A1 and *B1a have much higher frequencies in all other populations except the African population where *A3 has not been seen [15].

Previous studies on the role of GSTA1 promoter polymorphisms on the clearance and tolerability of busulfan treatments used mostly inferred haplotype/diplotype combinations [33], although we developed the molecular cloning strategies to experimentally determine phased haplotypes [15]. Determination of the diplotypes based on variant allele frequencies favors the *A1*B1a haplotype over the *A3*B2 one. According to our current results, most individuals are indeed likely to carry the *A1*B1a genotype, although the alternative should not be excluded. In terms of clinical impact, while the proper distinction between *A1*B1a and *A3*B2 might help refine the interpretation of the results and help understand the underlying transcriptional mechanisms, none of those diplotypes corresponds to an extreme metabolizer status associated with worse treatment outcomes [33].

The phasing was performed through direct sequencing of single DNA molecules corresponding to the *GSTA1* promoter region amplified by PCR. The technique used here improves the process of phasing substantially compared to classical methods based on molecular cloning or the analysis of related individuals. Phasing is also important for other pharmaco-genes such as TPMT, where the *1/*3A diplotype (normal metabolizer) or a *3B/*3C (slow metabolizer) cannot be discriminated using non-allele specific techniques [16]. A proper TPMT-based stratification of patients is capital as the assignment of a patient to a wrong metabolizer group could potentially lead to severe consequences, due to improper drug dosage [17]**.** The Oxford Nanopore technology had been successfully used, in the past, to phase haplotypes in the HLA region genes or the CYP2D6 gene [20, 21].

We also tried to probe the potential limitations and pitfalls of the technique used in this paper. The single nucleotide sequencing errors appear to be distributed randomly. The quality of the DNA product obtained by PCR amplification is expected to be the main driver in this type of haplotype phasing. If the PCR product is not fully extended during the initial reaction cycles, the formation of hybrid crossovers might occur. The earlier such hybrid cross-over appears in the reaction, the higher the final proportion of those hybrids will be in the PCR product. Therefore, cycling conditions, particularly extension time during the PCR amplification, might be important. However, we showed that in our conditions, neither an extensive reduction of the extension nor annealing time influenced the formation of hybrids. Regarding *GSTA1* promoter amplification, the haplotype rate remained stable at about 70% of true haplotypes even at distances above 600 bp. Moreover, reliable phasing being achievable at distances up to 600 bp, it is feasible to reconstruct correct haplotypes whenever the amplified region is sufficiently polymorphic. Optimal PCR primer design for the placement of phased SNPs in PCR products might also help as more central locations of PCR products have better sequencing coverage than the 3′ and 5′ ends. In addition to annealing and extension time, we analyzed the influence of the input DNA amount and PCR cycle number as other recent publications showed it can influence the hybrid haplotype formation [34–36]. Our results suggest that additional cycles after reaching the PCR plateau phase produce hybrid haplotypes and that additional cycles after the exponential phase of PCR should be avoided to minimize the generation of hybrid haplotypes. Initial DNA content did not influence the generation of hybrid haplotypes. Our current protocol, nevertheless, proved to be sufficiently robust to reliably phase GSTA1 promoter haplotypes. The techniques, sequencing results, and phasing quality in these publications are similar to ours [34–36].

In conclusion, we developed a simple and rapid strategy for allelic discrimination of *GSTA1* promoter haplotype that can be easily implemented in most laboratories and is better suited for use in routine clinical practice than currently used technologies. The technique proved robust for the *GSTA1* promoter and could be adapted to other haplotypes located in a genomic range that can be robustly amplified by PCR. Inter-SNP distances and frequencies are important determinants of correct phasing and can influence phasing strategy.

## Data Availability

Not applicable.

## References

[1] Caudle, K. E., Gammal, R. S., Whirl-Carrillo, M., Hoffman, J. M., Relling, M. V., & Klein, T. E. (2016). Evidence and resources to implement pharmacogenetic knowledge for precision medicine. *American Journal of Health System Pharmacy,**73*, 1977–1985. 27864205 10.2146/ajhp150977 PMC5117674

[2] O’Shea, J., Ledwidge, M., Gallagher, J., Keenan, C., & Ryan, C. (2022). Pharmacogenetic interventions to improve outcomes in patients with multimorbidity or prescribed polypharmacy: A systematic review. *The Pharmacogenomics Journal,**22*, 89–99. 35194175 10.1038/s41397-021-00260-6 PMC8975737

[3] Sadee, W., Wang, D., Hartmann, K., & Toland, A. E. (2023). Pharmacogenomics: Driving personalized medicine. *Pharmacological Reviews,**75*, 789–814. 36927888 10.1124/pharmrev.122.000810 PMC10289244

[4] Forrester, L. M., Hayes, J. D., Millis, R., Barnes, D., Harris, A. L., Schlager, J. J., Powis, G., & Wolf, C. R. (1990). Expression of glutathione S-transferases and cytochrome P450 in normal and tumor breast tissue. *Carcinogenesis,**11*, 2163–2170. 2265468 10.1093/carcin/11.12.2163

[5] Mannervik, B., Awasthi, Y. C., Board, P. G., Hayes, J. D., Di Ilio, C., Ketterer, B., Listowsky, I., Morgenstern, R., Muramatsu, M., Pearson, W. R., Pickett, C. B., Sato, K., Widersten, M., & Wolf, C. R. (1992). Nomenclature for human glutathione transferases. *The Biochemical Journal,**282*(Pt 1), 305–306. 1540145 10.1042/bj2820305 PMC1130923

[6] Coles, B. F., Morel, F., Rauch, C., Huber, W. W., Yang, M., Teitel, C. H., Green, B., Lang, N. P., & Kadlubar, F. F. (2001). Effect of polymorphism in the human glutathione S-transferase A1 promoter on hepatic GSTA1 and GSTA2 expression. *Pharmacogenetics,**11*, 663–669. 11692074 10.1097/00008571-200111000-00004

[7] Hayes, J. D., & Pulford, D. J. (1995). The glutathione S-transferase supergene family: Regulation of GST and the contribution of the isoenzymes to cancer chemoprotection and drug resistance. *Critical Reviews in Biochemistry and Molecular Biology,**30*, 445–600. 8770536 10.3109/10409239509083491

[8] Dirven, H. A., Dictus, E. L., Broeders, N. L., van Ommen, B., & van Bladeren, P. J. (1995). The role of human glutathione S-transferase isoenzymes in the formation of glutathione conjugates of the alkylating cytostatic drug thiotepa. *Cancer Research,**55*, 1701–1706. 7712478

[9] Ekhart, C., Doodeman, V. D., Rodenhuis, S., Smits, P. H., Beijnen, J. H., & Huitema, A. D. (2009). Polymorphisms of drug-metabolizing enzymes (GST, CYP2B6 and CYP3A) affect the pharmacokinetics of thiotepa and tepa. *British Journal of Clinical Pharmacology,**67*, 50–60. 19076156 10.1111/j.1365-2125.2008.03321.x PMC2668084

[10] Sargent, J. M., Williamson, C., Hall, A. G., Elgie, A. W., & Taylor, C. G. (1999). Evidence for the involvement of the glutathione pathway in drug resistance in AML. *Advances in Experimental Medicine and Biology,**457*, 205–209. 10500795 10.1007/978-1-4615-4811-9_22

[11] Wang, H. N., Zhu, X. Y., Zhu, Y., Xie, Q. H., Lai, L. Y., Zhao, M., Chen, Y. C., Xue, J., Hao, C. M., Gu, Y., & Lin, S. Y. (2015). The GSTA1 polymorphism and cyclophosphamide therapy outcomes in lupus nephritis patients. *Clinical Immunology,**160*, 342–348. 26222310 10.1016/j.clim.2015.07.010

[12] Czerwinski, M., Gibbs, J. P., & Slattery, J. T. (1996). Busulfan conjugation by glutathione S-transferases alpha, mu, and pi. *Drug Metabolism and Disposition,**24*, 1015–1019. 8886613

[13] Ansari, M., Huezo-Diaz, P., Rezgui, M. A., Marktel, S., Duval, M., Bittencourt, H., Cappelli, B., & Krajinovic, M. (2016). Influence of glutathione S-transferase gene polymorphisms on busulfan pharmacokinetics and outcome of hematopoietic stem-cell transplantation in thalassemia pediatric patients. *Bone Marrow Transplantation,**51*, 377–383. 26691424 10.1038/bmt.2015.321 PMC4777888

[14] Ansari, M., Theoret, Y., Rezgui, M. A., Peters, C., Mezziani, S., Desjean, C., Vachon, M. F., Champagne, M. A., Duval, M., Krajinovic, M., Bittencourt, H., Pediatric Disease Working Parties of the European Blood and Marrow Transplant Group. (2014). Association between busulfan exposure and outcome in children receiving intravenous busulfan before hematopoietic stem cell transplantation. *Therapeutic Drug Monitoring,**36*, 93–99. 24061446 10.1097/FTD.0b013e3182a04fc7

[15] Mlakar, V., Curtis, P. H., Armengol, M., Ythier, V., Dupanloup, I., Hassine, K. B., Lesne, L., Murr, R., Mlakar, S. J., Nava, T., & Ansari, M. (2021). The analysis of GSTA1 promoter genetic and functional diversity of human populations. *Science and Reports,**11*, 5038. 10.1038/s41598-021-83996-2 PMC7930039 33658540

[16] Brownstein, C. A., Margulies, D. M., & Manzi, S. F. (2014). Misinterpretation of TPMT by a DTC genetic testing company. *Clinical Pharmacology and Therapeutics,**95*, 598–600. 24714787 10.1038/clpt.2014.60

[17] Relling, M. V., Gardner, E. E., Sandborn, W. J., Schmiegelow, K., Pui, C. H., Yee, S. W., Stein, C. M., Carrillo, M., Evans, W. E., Klein, T. E., Clinical Pharmacogenetics Implementation Consortium. (2011). Clinical Pharmacogenetics Implementation Consortium guidelines for thiopurine methyltransferase genotype and thiopurine dosing. *Clinical Pharmacology & Therapeutics,**89*, 387–391. 21270794 10.1038/clpt.2010.320 PMC3098761

[18] Tait, B. D. (2022). The importance of establishing genetic phase in clinical medicine. *International Journal of Immunogenetics,**49*, 1–7. 34958529 10.1111/iji.12567

[19] Midha, M. K., Wu, M., & Chiu, K. P. (2019). Long-read sequencing in deciphering human genetics to a greater depth. *Human Genetics,**138*, 1201–1215. 31538236 10.1007/s00439-019-02064-y

[20] Lang, K., Surendranath, V., Quenzel, P., Schofl, G., Schmidt, A. H., & Lange, V. (2018). Full-Length HLA class I genotyping with the MinION nanopore sequencer. *Methods in Molecular Biology,**1802*, 155–162. 29858807 10.1007/978-1-4939-8546-3_10

[21] Liau, Y., Maggo, S., Miller, A. L., Pearson, J. F., Kennedy, M. A., & Cree, S. L. (2019). Nanopore sequencing of the pharmacogene CYP2D6 allows simultaneous haplotyping and detection of duplications. *Pharmacogenomics,**20*, 1033–1047. 31559921 10.2217/pgs-2019-0080

[22] Rhoads, A., & Au, K. F. (2015). PacBio sequencing and its applications. *Genomics, Proteomics & Bioinformatics,**13*, 278–289. 10.1016/j.gpb.2015.08.002 PMC4678779 26542840

[23] Wang, O., Cheng, X., Drmanac, R., & Peters, B. A. (2023). A simple cost-effective method for whole-genome sequencing, haplotyping, and assembly. *Methods in Molecular Biology,**2590*, 101–125. 36335495 10.1007/978-1-0716-2819-5_7

[24] 1000 Genomes Project Consortium, Auton, A., Brooks, L. D., Durbin, R. M., Garrison, E. P., Kang, H. M., Korbel, J. O., Marchini, J. L., McCarthy, S., McVean, G. A., & Abecasis, G. R. (2015). A global reference for human genetic variation. *Nature,**526*, 68–74. 26432245 10.1038/nature15393 PMC4750478

[25] Jurkovic Mlakar, S., Uppugunduri, S. C. R., Nava, T., Mlakar, V., Golay, H., Robin, S., Waespe, N., Rezgui, M. A., Chalandon, Y., Boelens, J. J., Bredius, R. G. M., Dalle, J. H., Peters, C., Corbacioglu, S., Bittencourt, H., Krajinovic, M., Ansari, M., Paediatric Diseases Working Party of the European Society for Blood and Marrow Transplantation. (2022). GSTM1 and GSTT1 double null genotypes determining cell fate and proliferation as potential risk factors of relapse in children with hematological malignancies after hematopoietic stem cell transplantation. *Journal of Cancer Research and Clinical Oncology,**148*, 71–86. 34499222 10.1007/s00432-021-03769-2 PMC8752561

[26] Steining, E., & Coin, L. (2022). Nanoq: Ultra-fast quality control for nanopore reads. *The Journal of Open Source Software,**7*, 1–6.35355633

[27] Andrews, S. (2010). FastQC: A quality control tool for high throughput sequence data. http://www.bioinformatics.babraham.ac.uk/projects/fastqc/

[28] Li, H. (2018). Minimap2: Pairwise alignment for nucleotide sequences. *Bioinformatics,**34*, 3094–3100. 29750242 10.1093/bioinformatics/bty191 PMC6137996

[29] Zheng, Z., Li, S., Su, J., Leung, A. W., Lam, T. W., & Luo, R. (2022). Symphonizing pileup and full-alignment for deep learning-based long-read variant calling. *Nature Computational Science,**2*, 797–803. 38177392 10.1038/s43588-022-00387-x

[30] Patterson, M., Marschall, T., Pisanti, N., van Iersel, L., Stougie, L., Klau, G. W., & Schonhuth, A. (2015). WhatsHap: Weighted haplotype assembly for future-generation sequencing reads. *Journal of Computational Biology,**22*, 498–509. 25658651 10.1089/cmb.2014.0157

[31] Danecek, P., Bonfield, J. K., Liddle, J., Marshall, J., Ohan, V., Pollard, M. O., Whitwham, A., Keane, T., McCarthy, S. A., Davies, R. M., & Li, H. (2021). Twelve years of SAMtools and BCFtools. *GigaScience*. 10.1093/gigascience/giab008 33590861 10.1093/gigascience/giab008 PMC7931819

[32] Team, R. C. (2022). *R: A language and environment for statistical computing*. R Foundation for Statistical Computing, Vienna, Austria. https://www.R-project.org/

[33] Ansari, M., Curtis, P. H., Uppugunduri, C. R. S., Rezgui, M. A., Nava, T., Mlakar, V., Lesne, L., Theoret, Y., Chalandon, Y., Dupuis, L. L., Schechter, T., Bartelink, I. H., Boelens, J. J., Bredius, R., Dalle, J. H., Azarnoush, S., Sedlacek, P., Lewis, V., Champagne, M., …, Krajinovic, M. (2017). GSTA1 diplotypes affect busulfan clearance and toxicity in children undergoing allogeneic hematopoietic stem cell transplantation: A multicenter study. *Oncotarget,**8*, 90852–90867. 10.18632/oncotarget.20310 PMC5710889 29207608

[34] Deserranno, K., Tilleman, L., Rubben, K., Deforce, D., & Van Nieuwerburgh, F. (2023). Targeted haplotyping in pharmacogenomics using Oxford Nanopore Technologies’ adaptive sampling. *Frontiers in Pharmacology,**14*, 1286764. 38026945 10.3389/fphar.2023.1286764 PMC10679755

[35] Maestri, S., Maturo, M. G., Cosentino, E., Marcolungo, L., Iadarola, B., Fortunati, E., Rossato, M., & Delledonne, M. (2020). A long-read sequencing approach for direct haplotype phasing in clinical settings. *International Journal of Molecular Sciences,**21*, 9177. 33271988 10.3390/ijms21239177 PMC7731377

[36] McClinton, B., Watson, C. M., Crinnion, L. A., McKibbin, M., Ali, M., Inglehearn, C. F., & Toomes, C. (2023). Haplotyping using long-range PCR and nanopore sequencing to phase variants: Lessons learned from the ABCA4 locus. *Laboratory Investigation,**103*, 100160. 37088464 10.1016/j.labinv.2023.100160

